# Is the process for selecting indigents to receive free care in Burkina Faso equitable?

**DOI:** 10.1186/1471-2458-14-1158

**Published:** 2014-11-07

**Authors:** Nicole Atchessi, Valéry Ridde, Maria-Victoria Zunzunégui

**Affiliations:** University of Montreal Hospital Research Centre (CRCHUM), Health Research Axis, Saint-Antoine Tower,850 Saint-Denis St., Suite S03.312, Montreal, Quebec H2X 0A9 Canada

**Keywords:** Indigent selection, User fee exemption, Equity, Sub-Saharan Africa

## Abstract

**Background:**

In Burkina Faso, patients are required to pay for healthcare. This constitutes a barrier to access for indigents, who are the most disadvantaged. User fee exemption systems have been created to facilitate their access. A community-based initiative was thus implemented in a rural region of Burkina Faso to select the worst-off and exempt them from user fees. The final selection was not based on pre-defined criteria, but rather on community members’ tacit knowledge of the villagers. The objective of this study was to analyze the equitable nature of this community-based selection process.

**Method:**

Based on a cross-sectional study carried out in 2010, we surveyed 1,687 indigents. The variables collected were those that determine healthcare use according to the Andersen-Newman model (1969): sociodemographic variables; income; occupation; access to financial, food or instrumental assistance; presence of chronic illness; and disabilities related to vision, muscle strength, or mobility. Bivariate analyses and logistic regression were performed.

**Results:**

User fee exemptions were given mainly to indigents who were widowed (OR = 1.40; CI 95% [1.10–1.78]), had no financial assistance from their household for healthcare (OR = 1.58; CI 95% [1.26–1.97], lived alone (OR = 1.28; CI 95% [1.01–1.63]), lived with their spouses, (OR = 2.00; CI 95% [1.35-2.96], had vision impairments (OR = 1.45; CI 95% [1.14–1.84]), or had poor muscle strength and good mobility (OR = 1.73; CI 95% [1.28–2.33]). The indigent selection was not determined by household income, self-reported chronic illness, or previous use of services.

**Conclusion:**

The community selection process took into account factors related to social vulnerability and functional limitations. However, we cannot affirm that the selection process was perfectly equitable, as it was very restrictive due to the limited budget available and the State’s lack of engagement in this matter. Exemption processes should be temporary solutions, and the State should make a commitment to move toward universal healthcare coverage.

## Background

In many sub-Saharan African countries, patients are still required to pay for healthcare at the point of service. In this context, indigents are the population most affected by the financial barrier to access to care [1, 2]. Some interventions have sought to facilitate their access by exempting them from user fees [3–6]. However, identifying the indigents who could benefit from these measures presents many challenges [7–9]. Few countries have specific indigence criteria, and the criteria and targeting processes used vary [3, 10–12]. Some countries opt for administrative targeting done by program managers, while others prefer participatory targeting carried out by members of the community [12]. It is difficult to know which approach is more effective, that is, which is able to identify those who are really indigent and those who are not, with indigents considered to be the poorest among the poor who are unable to pay for healthcare [7]. Effective targeting would require clear and precise criteria, numerous indicators to cover the various dimensions of indigence, and community-based selection backed up by the administrative process. For effective targeting, the local context of poverty must also be taken into account [3]. Effective indigent selection is essential to ensure those who really meet the definition of indigence benefit from the program intended for them. It also a means of ensuring that the sparse resources designated for the care of indigents in low-income countries are not squandered. For these reasons, it is relevant to analyze the equitable nature of the indigent selection process, since its purpose is to reduce inequalities of access to care. The equitable nature of the process refers to the social justice practised in the selection of indigents [13], such that the selection takes into account the fact that indigents have more serious characteristics and conditions of vulnerability than do others in this rural population. The characteristics of indigents that determine their vulnerability, such as poor health status, isolation, and lack of income, should thus be among the criteria applied in the selection process.

Very few studies have looked at the effectiveness of indigent targeting methods in Africa and their equity. Those studies were based essentially on income-related criteria, ignoring other key vulnerability factors such as age, social isolation, and health status [8, 14–16]. An evaluation of several indigent targeting methods in Ghana took into account geographic criteria as well as income-related criteria [16]. In Burkina Faso, a community-based targeting method was evaluated using indigents’ sociodemographic profile [14]. While these two studies went beyond financial and material criteria, they still did not fully explore all the dimensions of indigents’ vulnerability. Sociodemographic and economic criteria could be combined with different aspects of health status and social isolation to assess the equity of the targeting. The key contribution of our study to the current corpus of knowledge lies in the number and variety of vulnerability criteria used to measure the equitable nature of an indigent selection process in Burkina Faso.

In Burkina Faso, patients must pay for healthcare at the point of service. To facilitate access to care for indigents, the health centre management committees (COGESs) decided to allocate funds to cover user fee exemptions for the indigent. This user fee exemption program is an official, ongoing program that covers all services provided in the region’s public health centres, which are primary care services. However, it is not easy to identify who is indigent. We therefore conducted an action research project in the Ouargaye health district [3]. This district is described more fully later in this paper. The action research project involved organizing a participatory, community-based process to select indigents for exemption from user fees. Village health committees (VHCs) were set up in each village. They consisted of seven people from the community, including at least one religious leader; 51% of the members overall were women. In 2010, based on a definition of indigence that they develop consensually, and using a participatory process, the VHCs selected the people in their village whom they considered indigent.

Because it was not financially feasible to provide exemptions for all the indigents selected by the VHCs, the COGESs selected from those lists the people they considered the worst-off, to be given exemption cards. In fact, this exemption is funded by the COGESs from the profits generated from paid consultations and drug sales. This second level of selection was not based on pre-defined criteria, but rather, on the judgment of COGES members. Overall, 90% of COGES members were men and 10%, women. Indigents selected by the COGESs received an official card attesting to their status and giving them the right to be exempted from user fees. All recipients were informed of the benefits provided by this card. Indigents selected by the VHCs who were not subsequently also selected by the COGESs were not exempted from user fees. This selection method is restrictive because of the limited amount of resources allocated. It is a temporary solution, until such time as universal access to free healthcare can be implemented.

This selection process was intended to increase indigents’ use of health services. The model developed by Andersen and Newman [17] is particularly relevant for evaluating the selection process. It has been used mainly in studies on health services use, particularly among the elderly [18–20] and so is well-suited to our study, given that 75% of the indigents selected by the VHCs were over 50 years of age [3]. This model takes into account a number of variables, categorized as predisposing factors, enabling factors, and needs. Predisposing factors are sociodemographic variables that exist prior to the individual’s illness, e.g. age, sex, marital status. Enabling factors are the social and economic resources that either facilitate or impede the individual’s use of services. Needs correspond to the illnesses or disabilities for which the indigent person seeks healthcare. The Anderson-Newman model can also be used to analyze equity of access to healthcare [21].

The effectiveness of indigent targeting in Burkina Faso was evaluated in a 2007 pilot project of community-based selection in half the villages of the district. That study involved 184 indigents. The results showed that the selected indigents were the poorest and most vulnerable persons in the population, but that there was significant exclusion of the most vulnerable in terms of sociodemographic characteristics. Also, the analysis criteria used in that pilot project study were essentially related to financial and material assets [14], an important limitation found in most studies on targeting in Africa [6, 14, 15]. In the present original study, the analysis focuses on the results of the community-based selection process undertaken in all villages of the district (n = 26). Our study also adds to the knowledge base in this area by taking into account criteria related to age, sex, marital status, social isolation, and health status, which to our knowledge has not yet been done in Burkina Faso. We adopted this approach to be able to measure more precisely the equitable nature of the indigent selection process.

### Objective

The objective of this study was to analyze the equitable nature of the indigent selection process as conducted by the COGESs.

### Hypothesis

Given that COGES members come from the community, they are very likely to have a good knowledge of the socioeconomic situation and health status of members of their community, and their selection would be expected to focus on the most vulnerable indigents. Consequently, the hypothesis of this study was that the indigents selected by the COGESs would be the persons whom the community considers to be the most vulnerable, that is, women, the elderly, the worst-off, the most isolated, and those with the greatest number of functional disabilities.

## Method

### Research design and study population

This cross-sectional study was conducted in October 2010 in Ouargaye district, in the centre-east region of Burkina Faso. Of the country’s 13 regions, this is the fifth poorest, with about 55% of the population considered poor, having an annual income of less than 82,672 F CFA, or $200/year (USD). A healthcare consultation in rural health centres costs around 0.25 USD, to which is added drug costs. The attendance rate at healthcare centres in the centre-east region of Burkina Faso is 0.78 new consultations per year per person [22].

The study population consisted of all indigent persons aged 18 years and over selected by the VHCs (n = 2,093) in 2010. The list of the 2,093 indigent persons is available from the registers of the Social Action Department Administration of Ouargaye district.

The indigent persons were invited to participate in the survey by means of home visits. We were able to locate and interview 1,687 of these indigents, for a response rate of 80.6%. Of these, some (n = 751) were retained in the final selection carried out by the COGESs and received user fee exemption cards signed by the Ministry of Health and the Ministry of Social Action. Some indigents could not be found, either because they had died, moved away, or were temporarily absent. For some, the reasons were unknown.

Consent forms were completed with either the participant’s signature or digital fingerprint. Ethical approval for the study was obtained from the research ethics committees of the University of Montreal Hospital Research Centre and of Burkina Faso.

Data were collected by means of a questionnaire administered by trained surveyors in individual interviews conducted in participants’ homes. A portion of the questionnaire was addressed to the head of household and another portion to the indigent person.

### Variables

The variable of interest (outcome) in our study was possession of the card giving free access to healthcare services. This was a dichotomous variable.

The other variables in our study were those that determine the probability of service use according to Andersen and Newman, since the allocation of cards was intended to increase this probability. These variables were grouped into predisposing factors, enabling factors, and needs. All these variables were self-reported.

#### Predisposing factors

The predisposing factors were age, sex, level of education, and marital status. Age was self-reported and classified into three categories: under 50 years, 50 to 69 years, and over 69 years. Sex was a dichotomous variable. In rural Africa, educational level is represented by a two-category variable: educated and non-educated. We categorized marital status into four categories: single, married, divorced/widowed, and other, with the latter consisting of those who did not provide information about their marital status. This variable was transformed into a dichotomous variable (widowed and not widowed) for multivariate analyses, in order to expose the particular vulnerability associated with widowed marital status.

#### Enabling factors

In our study, the enabling factors were:

- The presence of an income-generating activity, if the indigent person carried out regular activities that generated a financial revenue; this was a dichotomous variable (yes/no).
- The income level of the indigent person’s household; this variable was measured using a proxy, household consumption, obtained by calculating the total per capita expenses of the indigent person’s household for the previous year for healthcare, food, schooling, and other various items; this was a continuous variable categorized into quintiles;
- Recourse to financial assistance within the household for healthcare, if the respondent received such support from other household members; this was a dichotomous variable (yes/no); not being able to obtain financial support from one’s household to purchase healthcare is an indicator of household poverty and also, by extension, of the indigent’s vulnerability.
- Recourse to external assistance to obtain food, if the respondent received such support from people outside the household; this was a dichotomous variable (yes/no); the fact that someone would need to ask for food outside the household is a sign of vulnerability.
- The availability of instrumental assistance if the person required help to carry out activities of daily living. When respondents answered yes to this question, they were asked whether there were persons in their entourage who provided such assistance; this variable had three categories: 1) no assistance needed; 2) assistance needed but not received; 3) assistance needed and provided by someone in the person’s entourage; the fact of not receiving instrumental assistance when it is needed is also a sign of vulnerability.
- Cohabitation: this variable measured respondents’ family support network. This variable considered the types of persons with whom the respondents lived and consisted of mutually exclusive categories: 1) living alone (no cohabitation); 2) cohabitation with children; 3) cohabitation with a spouse (perhaps also with children); and 4) cohabitation with parents, siblings, or friends/neighbours (perhaps also with children).

### Needs

The following needs were measured:

- The presence of a chronic illness, if the person was suffering from an illness that had persisted for more than six months; this was a dichotomous variable;
- The presence of any vision impairment, measured by considering both far and near vision, as was done by the World Health Organization in the *World Health Survey* [23]; far vision was considered impaired if during the previous 30 days the person had difficulty recognizing another known person at a distance of about 20 metres; near vision was considered to be impaired if during the previous 30 days the person had trouble recognizing something held in his or her hand. If respondents answered yes to either of these two questions, they were considered to have impaired vision. In all other cases, they were considered not to have impaired vision. Thus, this variable was dichotomous: presence or absence of impaired vision.
- The presence of functional physical limitations, explored based on the work of Nagi [24] and Guralnik [25]. All of these variables were dichotomous:

- Limitation in arm muscle strength, defined as difficulty in lifting or carrying weights greater than 5 kg, such as a sack of millet (in the local context, a sack of millet refers to a quantity of wheat weighing around 5 kg) or a bucket of water [24];
- Limitation of mobility, defined as difficulty in walking a distance of 400 metres [25];
- Limitation of fine finger movements, defined as difficulty in grasping or manipulating small objects with one’s fingers [24] .
- Limitation in arm abduction movements, defined as difficulty in raising one’s arms above one’s head [24].

In the multivariate analyses, the variables “limitation in arm muscle strength” and “limitation of mobility” were combined into a single variable indicating physical disability. The new combined variable included three categories: 1) poor mobility; 2) good mobility and poor muscle strength; and 3) good mobility and good strength.

#### Use of modern healthcare services

This variable was measured to capture indigents’ use of services *before* the cards were given out. The indigents were questioned about their use of modern healthcare services over the **previous six months**. This was a dichotomous variable.

#### Analyses

To begin, using IBM® SPSS 19 software, bivariate analyses were carried out between the variable “possession of a card” and all the other variables. Then logistic regression was performed focusing on the variables that were significant (p <0.25) in the bivariate analyses. In the first stage, only the variables corresponding to predisposing factors were included in the model. In the second stage, the predisposing factors that remained significantly associated (p <0.05) with service utilization in the first stage were kept in the model, and the variables corresponding to enabling factors were added. In the third stage, the variables corresponding to predisposing factors and enabling factors that remained significant (p <0.05) in the second stage were kept in the model, and the variables corresponding to need were added. The final model took into account the significant variables (p <0.05). However, to facilitate the understanding of the results, certain key demographic variables, such as age and sex, were kept in the model even if they did not remain significant.

### Results

#### Bivariate analyses

Table 1 presents the characteristics of all the indigents and the proportions of both those who received a user fee exemption card in 2010 and who did not.

**Table 1:** **Characteristics of indigents given and not given exemption cards in 2010**

			N	Indigents selected by VHCs (not given cards)	Indigents selected by COGESs (given cards)	
			N	%	%	p
**Predisposing factors**	**Sex**	Male	826	48.8	49.1	0.92
		Female	861	51.2	50.9	
	**Age group (years)**	< 50	412	27.9	20.0	0.0001
		50 to 69	687	41.6	39.7	
		> 69	588	30.5	40.3	
	**Marital status**	Single	181	8.7	13.5	0.0001
		Married	702	47.1	34.7	
		Widowed/divorced	728	39.8	47.4	
		Other	76	4.6	4.4	
**Enabling factors**	**Income-generating activity**	No	1595	93.2	96.3	0.007
		Yes	92	6.8	3.7	
	**Household income**	Quintile1	338	21.7	17.9	0.048
		Quintile2	337	19.0	21.2	
		Quintile3	338	18.0	22.6	
		Quintile4	338	20.4	19.6	
		Quintile5	336	20.9	18.7	
	**Financial assistance from household for healthcare**	No	1182	33.0	26.0	0.002
		Yes	505	67.0	74.0	
	**Outside assistance for food**	Non	1404	86.4	79.3	0.0001
		Oui	283	13.6	20.7	
	**Instrumental assistance**	Not needed	987	62.8	58.5	0.0001
		No	164	8.2	9.7	
		Yes	536	29.0	31.8	
	**Cohabitation with**	Alone	865	50.0	53.3	0.006
		Siblings/parents/friends	155	8.5	10.0	
		Spouse	158	8.2	10.8	
		Children	506	33.3	25.9	
**Needs**	**Chronic illness**	No	911	56.5	50.9	0.024
		Yes	776	43.5	49.1	
	**Vision impairment**	No	891	59.0	45.1	0.0001
		Yes	796	41.0	54.9	
	**Limitations of mobility**	No	1030	64.0	57.4	0.004
		Yes	657	36.0	42.6	
	**Reduced muscle strength**	No	765	51.5	37.7	0.0001
		Yes	922	41.5	62.3	
	**Difficulties with fine finger movements**	No	1311	80.7	74.0	0.001
		Yes	376	19.3	26.0	
	**Difficulties with arm abduction**	No	1392	85.1	79.3	0.002
		Yes	295	14.9	20.7	
**Utilization**	**No**		1224	27.1	27.9	0.7
	**Yes**		463	72.9	72.1	

#### Predisposing factors

The indigents who benefited the most from the exemption cards were those over 69 years of age (p = 0.0001), single persons, and widows/widowers (p = 0.0001). Women and men received cards in nearly equal proportions.

#### Enabling factors

There was an association between all the enabling factors and the allocation of exemption cards. The indigents who received exemption cards were, in the majority of cases, those in the most disadvantaged situations. They were those with no income-generating activity (p = 0.007), those who turned to sources outside of their household for food (p = 0.0001), those with no financial support from their household for obtaining healthcare services (p = 0.002), and those who needed instrumental assistance in their activities of daily living but received none from their entourage (p = 0.0001). Most of the indigents who received an exemption card belonged to households whose incomes corresponded to the second and third quintiles (p = 0.048). However, some indigents living in less seriously disadvantaged conditions than others also received exemption cards. These were people living with a spouse (p = 0.006), whereas very few of those living without a spouse and with children received cards.

#### Needs: health and functional disabilities

The probability of receiving a card was higher among those presenting health needs. These were persons with chronic illnesses (p = 0.024), with visual impairments (p = 0.0001), and/or with physical disabilities in terms of mobility (p = 0.004) and muscle strength (p = 0.0001).

Furthermore, card allocation was not associated with healthcare service use in the preceding six months.

#### Needs: by sex

The groups receiving exemption cards were the same among men and women, except in the case of fine finger movement limitations, where women received more.

#### Multivariate analysis

The adjusted odds ratios (OR) for the factors associated with the allocation of exemption cards to indigents are presented in Table 2.

**Table 2:** **Multivariate-adjusted odds ratios for the allocation of exemption cards to indigents**

Variables			Adjusted OR	CI 95%
**Predisposing factors**	**Sex**	**Male (Ref)**		
		Female	0.98	[0.78–1.23]
	**Age group (years)**	**< 49 (Ref)**		
		50 to 69	1.14	[0.83–1.57]
		> 69	1.02	[0.77–1.34]
	**Marital status**	**Not widowed (Ref)**		
		Widowed	1.40*	[1.10–1.78]
**Enabling factors**	**Financial assistance from household for healthcare**	**Yes (Ref)**		
		No	1.58***	[1.26–1.97]
	**Instrumental assistance**	**Not needed (Ref)**		
		No	1.15	[0.86–1.53]
		Yes	1.18	[0.80-1.75]
	**Cohabitation with**	**Children (Ref)**		
		Alone	1.28*	[1.01–1.63]
		Siblings/parents/friends	1.38	[0.95–2.01]
		Spouse	2.00***	[1.35–2.96]
**Needs**	**Chronic illness**	**No (Ref)**		
		Yes	1.12	[0.90–1.38]
	**Vision impairment**	**No (Ref)**		
		Yes	1.45**	[1.14–1.84]
	**Mobility and muscle strength**	**Good mobility/Good strength (Ref)**		
		Poor mobility	1.09	[0.80–1.50]
		Good mobility/Poor strength	1.73***	[1.28–2.33]
**Utilization**	**No**	**(Ref)**		
	**Yes**		0.92	[0.73–1.15]

*p <0.05; **p <0.01; ***p <0.001.

The allocation of exemption cards was significantly associated with widowed marital status (OR = 1.40; CI 95% [1.10–1.78]), with not receiving financial assistance from within the household to obtain healthcare services (OR = 1.58; CI 95% [1.26–1.97]), and with living alone (OR = 1.28; CI 95% [1.01–1.63]) or cohabiting with a spouse (with or without children) (OR = 2.00; CI 95% [1.35–2.96]). The same was true for indigents with vision impairments (OR = 1.45 CI 95% [1.14–1.84]) and those with good mobility and poor muscle strength (OR = 1.73; CI 95% [1.28–2.33]).

The allocation of cards was not associated with age, with needing instrumental assistance with activities of daily living, with self-reported chronic illnesses, or with prior use of healthcare services.

### Discussion

This study showed that the indigents selected by COGESs to receive exemption cards were, for the most part, those living in the most disadvantaged conditions. They were widows/widowers, those without financial assistance from their household to obtain care, those living alone, and those with vision impairments. The indigents selected by COGESs who lived in less seriously disadvantaged conditions than others and still received the card were mostly those living with their spouses and those with poor muscle strength and good mobility. That being said, the results overall showed that it was the indigents selected by the COGESs who were living in the most extremely disadvantaged conditions who benefited from the exemption program.

### The most vulnerable indigents were selected by COGESs

An indigent person’s inability to receive financial assistance from his or her household to obtain care reflects the financial difficulties of that household. This means the indigents selected by COGESs to receive the cards were those living in households with the fewest financial resources. These results are consistent with a study done on a smaller scale in the same region, in which the selected indigents were those whose households had the fewest financial and material resources [14]. A study conducted in Ghana in 2010, in which household well-being was considered in indigent selection, was not effective in identifying the most disadvantaged in regions where the poverty rate is high [16]. On the other hand, an evaluation conducted in the Nouna region of Burkina Faso showed that a selection process similar to the one used in Ghana was able to target the most vulnerable indigents [6].

The greatest beneficiaries of the exemption cards allocated by the COGESs were widows and widowers. This result is also consistent with the smaller-scale study done in the same district [14]. This is an equitable aspect of this selection method. In fact, widowed persons are more vulnerable than others, especially if they are elderly, as in the present study. In previous studies, criteria related to marital status were not used in indigent selection. The fact that most indigents living alone were also selected by COGESs is in keeping with this line of thinking. Isolation is a negative factor for mental health. Persons living in isolation often have limited moral support and receive little assistance in activities of daily living. They are also more inclined to have precarious mental health [26–29]. They are very likely to find it more difficult to get to a health centre when in need and to obtain financial assistance from family or friends. For these persons, removing the financial barrier could foster their use of healthcare services.

Most of the indigents with vision impairments who were selected by VHCs were also selected by the COGESs. In this study, vision impairments ranged from blurred vision to total blindness. *World Health Survey* results from 70 countries showed that vision impairments are most serious in low-income countries [30]. Moreover, these problems are more prevalent among the elderly, women, and people of disadvantaged socioeconomic status [30, 31]. Vision impairments can result from infectious diseases or complications of chronic illnesses such as diabetes or hypertension [32]. Considering vision impairments as a criterion in indigent selection is an equitable aspect of the selection method.

### Indigents living in less seriously disadvantaged conditions than others were selected by the COGESs

Most of the indigents with good mobility and poor muscle strength were selected by the COGESs, which was not the case for those with poor mobility. In the smaller-scale study, the indigents selected were those with the most physical and mental disabilities [14]. However, that earlier study was not sufficiently specific in describing the types of physical disabilities. The more detailed measurement of disabilities in the present study highlights a shortcoming in the selection method. Indigents with poor mobility have more difficulty getting around and consequently are less able to go to a health centre when necessary. A spatial analysis of this same population of indigents showed that those who were selected were the ones living nearest to the health centres [33]. All these results suggest the COGESs probably gave preference to people who had a higher probability of being able to get to the health centre and who would thereby benefit more fully from the user fee exemption program [33]. Indeed, it may be that removing the financial barrier is not, on its own, enough to increase service use among people with poor mobility. Even if the indigents selected in this category were not the most vulnerable, they were nevertheless afflicted with physical disabilities in terms of poor muscle strength. The fact that COGESs took into consideration physical disabilities when selecting indigents is an encouraging result, given that populations in sub-Saharan Africa, both old and young, often suffer from such disabilities [34, 35].

Most of the indigents living with their spouses were selected by the COGESs, whereas those living with children but without spouses were not selected in such numbers. This aspect of the selection process does not appear equitable, given that children in a household constitute a burden that is both financial and material. In fact, having a child under the age of five in a household was used as a proxy for poverty in the study conducted in the neighbouring country of Ghana [16]. It may be that, paradoxically, the COGESs considered having children to be an asset, a source of income and support in farming activities, as is often expressed in the local culture [36]. Nevertheless, to be equitable, such a selection process should instead regard children in a household as being a financial burden, which would more appropriately result in the selection of a majority of indigents living with children.

### The indigent selection process did not take into account certain important factors

The selection process was not gender-focused. Men and women were selected by the COGESs in approximately equal numbers. This may be due to the fact that the overall composition of the village selection committees doing the initial selections included equal numbers of men and women, unlike the COGESs. Even though the COGESs did not have the same gender parity in their composition that characterized the VHCs, they nevertheless maintained gender parity in the indigent selection. Thus, the fact that there was no association between card allocation and gender may be due to gender parity in the VHCs’ composition. This parity was subsequently respected by the COGESs in their selection.

Age, which appeared to be a significant factor in the bivariate analyses, lost its significance in the multivariate analyses. Older persons were given cards only when they presented other vulnerability criteria. Advanced age in itself was not considered a vulnerability criterion. This result is inconsistent with the smaller-scale study, which found that advanced age was a selection criterion [14]. This difference in results may be due to the type of analysis performed. In the smaller-scale study, comparative ANOVA analyses were done, whereas in the present study, multivariate analyses were able to control for confounding variables in the association between age and chronic illnesses such as widowhood, and disabilities.

Needing assistance in activities of daily living and having a chronic illness were not criteria in the COGESs’ selection of indigents. It may be that the COGESs have limited knowledge of the chronic illnesses afflicting the members of their communities. Some chronic illnesses such as hypertension are, in fact, not very visible. The smaller-scale study reported that cards were given to patients with more serious illnesses [14]. However, that study did not specify what types of illnesses or disabilities were taken into account.

Finally, prior use of healthcare services did not influence the COGESs’ selection of indigents for the exemption program. It might have been expected that those who had used services the least over the previous six months would benefit the most from receiving a card.

That being said, it is important to note from the outset that exemption programs such as the one studied here should be understood as temporary stopgap measures until such time as the State commits to providing universal healthcare coverage. The fact is that, while these solutions allow some of the poorest to access healthcare services, they still cannot make the healthcare system entirely equitable. These exemption systems are seriously limited in terms of being able to satisfy this objective. Decision-makers need to focus on the longer term and move toward ensuring healthcare access for all, and not only for indigents. Burkina Faso’s policies are still far from reaching this goal, but they are moving in the right direction. In 2007 the State implemented a national subsidy for facility-based deliveries, and since 2012 there has been an internal document proposing total fees exemptions for children and pregnant women.

### Strengths and limitations of the study

The cross-sectional design appears to have been appropriate for the research question. The multivariate analyses made it possible to measure each variable’s contribution to the analysis model while controlling for covariables. These methodological elements were strengths of this study, as compared with the smaller-scale study conducted in 2007 [14]. However, this study did have certain limitations.

One limitation had to do with measuring the indigents’ ages. Most did not know their real age and had no official documentation of it, so ages may have been under- or over-estimated.

Cognitive function would have been an important variable to measure, as it could have provided information on the cognitive limitations of the indigents selected [35]. This was not possible because some questions in the measurement instrument were not applicable, for sociocultural reasons.

In estimating household expenses, it would have been helpful to have information on food grown by the household for their own consumption.

All variables on health status and disabilities were self-reported, and as such, it is possible that some of this information was not entirely accurate.

We did not compare the indigents selected by the VHCs with the general population, as such data were not available in the region. It is possible that some of those selected were not indigents.

As defined in our article, the concept of equity in the selection process is based on qualitative concepts. While it is possible to identify degrees of indigence based on the vulnerability characteristics we measured, it is difficult to identify an indigence threshold. Establishing a threshold could lead to classification errors, which we would be unable to assess in the absence of a gold standard.

### Conclusion

To our knowledge, this is the only study on targeting the worst-off that has taken into account several other factors, such as age, health status, isolation, and income-related criteria. It is also one of only a small handful that have used multivariate analyses to evaluate the independent contributions of the variables to the allocation of exemption cards. The results showed that selection processes carried out in all the villages of Ouargaye district in Burkina Faso by COGESs most often led to the selection of the most vulnerable indigents. Even though the real indigents were selected, we cannot affirm that the selection process was equitable, given that the COGESs’ selections were guided by budget restrictions and that those indigents were selected from among a wider population of indigents. Nevertheless, we know for certain that those who were selected really were indigents. In fact, it was the indigents who were living in the most extreme conditions of vulnerability who benefited from the user fee exemptions, and so to some extent this is a positive outcome. Exemption programs such as the one studied here should be understood as temporary stopgap measures until such time as the State commits to providing universal healthcare coverage.
